# Supplementary material for: Influence of Voluntary Contraction Level, Test Stimulus Intensity and Normalization Procedures on the Evaluation of Short-Interval Intracortical Inhibition
Source: Brain Sci. 2020 Jul 8;10(7):433. doi: 10.3390/brainsci10070433 (PMC7407177; doi:10.3390/brainsci10070433)
Supplement: Supplementary file 1 [file brainsci-10-00433-s001.pdf]

Supplementary Materials

Table S1. Regression analysis between MEP_{test}/Mmax ratio (independent variable) and $SICI_{Mmax}$ (dependent variable) estimated individually for each subject with TS intensity set at 120%MT (left) and 130%MT (right).

**Regression Analysis (120 % MT)**					**Regression Analysis (130 % MT)**				
**Subject**	**Slope**	**Intercept**	**R^2**	***p* Value**	**Subject**	**Slope**	**Intercept**	**R^2**	***p* Value**
S01	−0.548	1.288	0.946	0.027	S01	−0.536	−1.650	0.903	0.050
S02	−0.689	−0.124	0.916	0.043	S02	−0.847	0.281	0.998	0.001
S03	−0.511	−0.990	0.986	0.007	S03	−0.624	−0.384	0.835	0.086
S04	−0.613	−0.459	0.933	0.034	S04	−0.491	−2.349	0.879	0.062
S05	−0.725	0.544	0.988	0.006	S05	−0.435	−0.808	0.870	0.068
S06	−0.694	1.472	0.990	0.005	S06	−0.661	−1.159	0.882	0.061
S07	−0.712	−1.530	0.995	0.003	S07	−0.730	−2.509	0.884	0.060
S08	−0.466	−8.373	0.775	0.120	S08	−0.488	−2.200	0.994	0.003
S09	−0.561	−2.754	0.778	0.118	S09	−0.675	−0.422	0.992	0.004
S10	−0.552	−1.895	0.999	0.001	S10	−0.428	−4.844	0.930	0.036
S11	−0.649	1.006	0.981	0.010	S11	−0.570	−0.737	0.945	0.028
S12	−0.385	−3.212	0.931	0.035	S12	−0.507	−3.436	0.925	0.038
S13	−0.510	−4.064	0.964	0.018	S13	−0.322	−5.223	0.943	0.029
S14	−0.419	−0.920	0.841	0.083	S14	−0.371	−2.589	0.968	0.016
S15	−0.672	−1.781	0.935	0.033	S15	−0.674	−1.148	0.935	0.033
Mean	−0.580	−1.453	0.931		Mean	−0.557	−1.945	0.925	
SD	0.109	2.524	0.075		SD	0.144	1.607	0.050	
